# Use of dementia care mapping in the care for older people with intellectual disabilities: A mixed‐method study

**DOI:** 10.1111/jar.12794

**Published:** 2020-08-18

**Authors:** Feija D. Schaap, Geke J. Dijkstra, Sijmen A. Reijneveld, Evelyn J. Finnema

**Affiliations:** ^1^ Research Group Living, Wellbeing and Care for Older People NHL Stenden University of Applied Sciences Leeuwarden The Netherlands; ^2^ Department of Health Sciences, Community and Occupational Medicine University Medical Center Groningen University of Groningen Groningen The Netherlands; ^3^ Department of Health Sciences, Applied Health Research University Medical Center Groningen University of Groningen Groningen The Netherlands; ^4^ Department of Health Sciences, Nursing Research University Medical Center Groningen University of Groningen Groningen The Netherlands

**Keywords:** ageing, dementia, Dementia Care Mapping, intellectual disability, mixed‐methods, person‐centred care

## Abstract

**Background:**

The ageing of people with intellectual disabilities, with associated morbidity like dementia, calls for new types of care. Person‐centred methods may support care staff in providing this, an example being Dementia Care Mapping (DCM). DCM has been shown to be feasible in ID‐care. We examined the experiences of ID‐professionals in using DCM.

**Methods:**

We performed a mixed‐methods study, using quantitative data from care staff (*N* = 136) and qualitative data (focus‐groups, individual interviews) from care staff, group home managers and DCM‐in‐intellectual disabilities mappers (*N* = 53).

**Results:**

DCM provided new insights into the behaviours of clients, enabled professional reflection and gave new knowledge and skills regarding dementia and person‐centred care. Appreciation of DCM further increased after the second cycle of application.

**Conclusion:**

DCM is perceived as valuable in ID‐care. Further assessment is needed of its effectiveness in ID‐care with respect to quality of care, staff‐client interactions and job performance.

## 1 INTRODUCTION

The increasing number of older people with intellectual disabilities and associated diseases such as dementia calls for new types of care (Haveman et al., 2010; Heller & Sorensen, 2013; McCarron, Swinburne, & McCallion, 2012; Patja, Iivanainen, Vesala, Oksanen, & Ruoppila, 2000). Several studies have outlined the difficulties encountered by ID‐care staff in dealing with psychosocial age‐related issues of their clients, such as dementia (Bowers, Webber, & Bigby, 2014; Webber, Bowers, & Bigby, 2016). Although staff have a strong commitment to help residents to remain in their own homes, they often lack knowledge and skills regarding older people with intellectual disabilities, in particular, those with dementia (Cleary & Doody, 2017a, 2017b; Holst, Johansson, & Ahlström, 2018; Iacono, Bigby, Carling‐Jenkins, & Torr, 2014; Wilkinson, Kerr, Cunningham, & Rae, 2004). This lack impedes adequate care (Bigby, 2008; Iacono et al., 2014; Janicki, Dalton, McCallion, Baxley, & Zendell, 2005; Watchman, 2008). Therefore, ID‐care staff have expressed a need for methods, knowledge and skills to support their older clients, especially in case of dementia (Cleary & Doodey, 2017b; Iacono et al., 2014; Janicki, 2011; Schaap, Fokkens, Dijkstra, Reijneveld, & Finnema, 2018; Webber et al., 2016). Such guidance can be found within person‐centred methods, derived from regular dementia care (Bickenbach et al., 2012; Campens et al., 2017; Cleary & Doody, 2017a, 2017b; Wilkinson et al., 2004).

In caring for older people with intellectual disabilities, person‐centred methods are promising and may contribute to the shift from task‐focused to more supportive care (Brown et al., 2016; Cleary & Doody, 2017a, 2017b; Ouellette‐Kuntz et al., 2019; Van der Meer, Nieboer, Finkenflügel, & Cramm, 2017). Person‐centred care puts the person at the centre of care services, rather than the disease (WHO, 2020). Person‐centred care evolved from the field of dementia care and is strongly connected to Tom Kitwood's concept of personhood in dementia (Kadri et al., 2018; Kitwood, 1998). Personhood refers to the relational aspects of being human, and the importance of being in an inclusive psychosocial environment where people are recognized as a person with a unique personality and life history who need a unique approach (Brooker, 2003; Brooker, Woolley, & Lee, 2007; Edvardsson, Winblad, & Sandman, 2008; Kitwood, 1992, 1998). Kitwood's view model highlights the relational components of personhood, engaging both the “cared for” and the “carer” in its construction and maintenance (Kadri et al., 2018). Kitwood stressed that the nature of cognitive and functional impairments associated with dementia (e.g., language and executive function) makes it difficult for people with dementia to meet their own needs (Kitwood, 1992, 1997; Willemse et al., 2015). To meet these psychological needs, person‐centred care provided by professional staff should comply with four major elements summarized in Brooker's VIPS framework: (a) an assertion of the absolute value of all human lives, regardless of age or cognitive ability; (b) an individualized approach, recognizing uniqueness of the person; (c) an understanding of the world from the perspective of the person; (d) a positive social psychology, enabling the person to experience relative well‐being (Brooker et al., 2007; Røsvik, Brooker, Mjorud, & Kirkevold, 2013; Røsvik, Kirkevold, Engedal, Brooker, & Kirkevold, 2011). Person‐centred care can yield more effective interactions between clients and care professionals (Brownie & Nancarrow, 2013; Tay et al., 2018; Van der Meer et al., 2017), and better collaboration of staff in coordination of care (Edvardsson et al., 2008; Rathert, Wyrwich, & Boren, 2013). In ID‐care, however, person‐centred methods, usually derived directly from regular dementia care, are often used in an unsystematic way (Fokkens, IJsbrandij, & Jansen, 2016; Schaap, Finnema, Stewart, Dijkstra, & Reijneveld, 2019a), even though previous research has strongly indicated that they should be customized to be successful (Hodes, Meppelder, Schuengel, & Kef, 2014; Vlaskamp, Hiemstra, & Wiersma, 2007).

One such person‐centred method, Dementia Care Mapping (DCM), designed to support staff in their daily care for people with dementia, has also been adapted to ID‐care (Schaap, Dijkstra, Finnema, & Reijneveld, 2018; Schaap, Fokkens, et al., 2018). DCM has characteristics that enhance innovation in ID‐care; it is a structured psychosocial method, based on the principles of person‐centred care, aimed at increasing the quality of care (Finnamore & Lord, 2007; Jaycock, Persaud, & Johnson, 2006; Persaud & Jaycock, 2001; Van de Ven et al., 2013). DCM is an intensive observational tool. It is used within a cycle of practice development for staff care settings, and is simultaneously an approach to achieve and embed person‐centred care for people with dementia (Surr et al., 2016). DCM is a cyclic method, consisting of a structured observation of six hours, followed by feedback to the whole care‐team, and action planning (see Box 1). This helps staff to reflect on their routine behaviour and interactions in daily care, thereby improving care from a client‐centred perspective. In small‐scale, qualitative studies, DCM has shown to be feasible and promising in supporting staff in caring for older people with intellectual disabilities, whether or not with dementia, in the United Kingdom and the Netherlands (Finnamore & Lord, 2007; Jaycock et al., 2006; Persaud & Jaycock, 2001; Schaap, Dijkstra, et al., 2018; Schaap, Fokkens, et al., 2018).

Box 1 **Dementia Care Mapping in people with intellectual disabilities: person‐centred care in action**

Dementia Care Mapping (DCM) is an intervention developed by the Dementia Research Group at Bradford University to improve the quality and effectiveness of care from the perspective of people with dementia (Brooker & Surr, 2005). It is based on Kitwood's social–psychological theory of personhood in dementia.(Kitwood, 1992) DCM was designed as observational tool to develop person‐centred care for people with dementia in nursing homes (Van de Ven et al., 2013). Person‐centred dementia care can be specified as: valuing people with dementia (V); using an individual approach that recognises the uniqueness of the person (I); making an effort to understand the world from the perspective of the person (P); and providing a supportive social environment (S) (Brooker et al., 2007). DCM has three main components:

***A: Mappers’ training in DCM***

A staff member receives training to become a certified DCM mapper. A basic DCM mapping course includes 4 days of basic concepts and skills. To participate in research, a mapper must achieve the level of advanced mapper. Required for this is a three‐day course focused on the background and theory of DCM, and person‐centred care. An advanced DCM mapper can observe (map) care with an inter‐reliability score of ≥0.8, report the observation, provide feedback and instruct staff in drawing up action plans (Van de Ven et al., 2013).

***B: Organizational introductory briefing***

Before the mapping (systematic observation of the actual care) takes place, the basic principles of DCM and person‐centred care are explained to the complete staff of a group home, to ensure endorsement and appropriate implementation (Van de Ven et al., 2013).

***C: DCM cycle: observations‐feedback‐action plan***

The introductory DCM organizational briefing day is followed by a DCM‐cycle, consisting of:

***Observation, analysis and report*.** A mapper observes four to six residents in communal areas for 4 to 6 hr. For each 5‐min time frame, a code is noted to record what happened with each resident and the associated behaviour of the staff. The DCM coding protocol contains 23 behavioural category codes (BCCs), well/ill‐being (WIB) values of clients, and personal detractions (PDs) and personal enhancers (PEs) in staff–client interactions (Brooker & Surr, 2005). PDs and PEs refer to staff behaviour and are often related to the WIB values in the interpretation of observations. After analysis the observations are included in a written report.

***Feedback*.** The results of the mapping are communicated verbally to the staff. The purpose of this feedback is to discuss and gain insight into residents’ behaviour in the context of both their lives and the care (Brooker & Surr, 2005). The feedback is complemented with knowledge of dementia and person‐centred care. Feedback is presented in a non‐threatening way and is intended to enhance staff awareness of their own and residents’ behaviour and of staff‐resident interactions, thereby motivating them to improve their competencies, performance and interactions (Van de Ven et al., 2013). The feedback is supported by the written report.

***Action plans*.** Based on observation and feedback, the staff draw up action plans to improve care at individual and group levels. Action plans are tools to implement theoretical knowledge of dementia and the principles of person‐centred care in daily practice, and to increase uniformity of care.

Quantitative findings on the effects of DCM in ID‐care are, however, conflicting. On the one hand, studies did not show positive effects of DCM, neither on the quality of life of older people with intellectual disabilities (Schaap et al., 2019a) nor on the job satisfaction of ID‐care staff (Schaap, Finnema, Stewart, Dijkstra, & Reijneveld, 2019b). On the other hand, staff reported that DCM provided adequate psychosocial methods and approaches to care for older people with intellectual disabilities (Schaap, Dijkstra, et al., 2018; Schaap, Fokkens, et al., 2018). Such contradictory findings regarding the use of DCM have also been reported for dementia care (Barbosa, Lord, Blighe, & Mountain, 2017; Chenoweth et al., 2009; Dichter et al., 2015; Rokstad et al., 2013; Van de Ven et al., 2013). Further assessment of the reasons underlying these contradictory findings may contribute to a better understanding of DCM and its use in ID‐care. The aim of this study is therefore to examine the experiences and opinions of staff and group home managers in the use of DCM in ID‐care.

## 2 METHODS

### 2.1 Study design

To obtain information from ID‐care professionals on their experiences in using DCM‐in‐ID, we used quantitative and qualitative methods after each of two DCM‐cycles. The quantitative method involved collecting data on the opinions of staff members after each application of DCM, via questions in a follow‐up questionnaire for a quasi‐experimental study on DCM. The qualitative method involved collecting in‐depth data from all ID‐care staff after each DCM‐cycle, using focus‐group discussions and face‐to‐face interviews. We performed the design, analysis and reporting according to the COREQ checklist (Tong, Sainsbury, & Craig, 2007).

The study was performed in accordance with the Helsinki Declaration, and informed consent was obtained from all participants (World Medical Association, 2013). The Medical Ethical Committee of the University Medical Center Groningen approved this study according to the ethical regulations of the Medical Research Involving Human Subjects Act (decision M13.146536).

### 2.2 Dementia care mapping in intellectual disability‐care

DCM‐in‐ID consists of a structured observation of six hours, followed by feedback of this observation to the whole care‐team, and then action planning (see Box 1). First, based on the findings of a feasibility study of DCM in ID‐care, in each group home four older clients were mapped simultaneously by a mapper not affiliated with the group home, for a total of six hours distributed across two or three separate periods (Schaap, Fokkens, et al., 2018).

**Figure 1:**
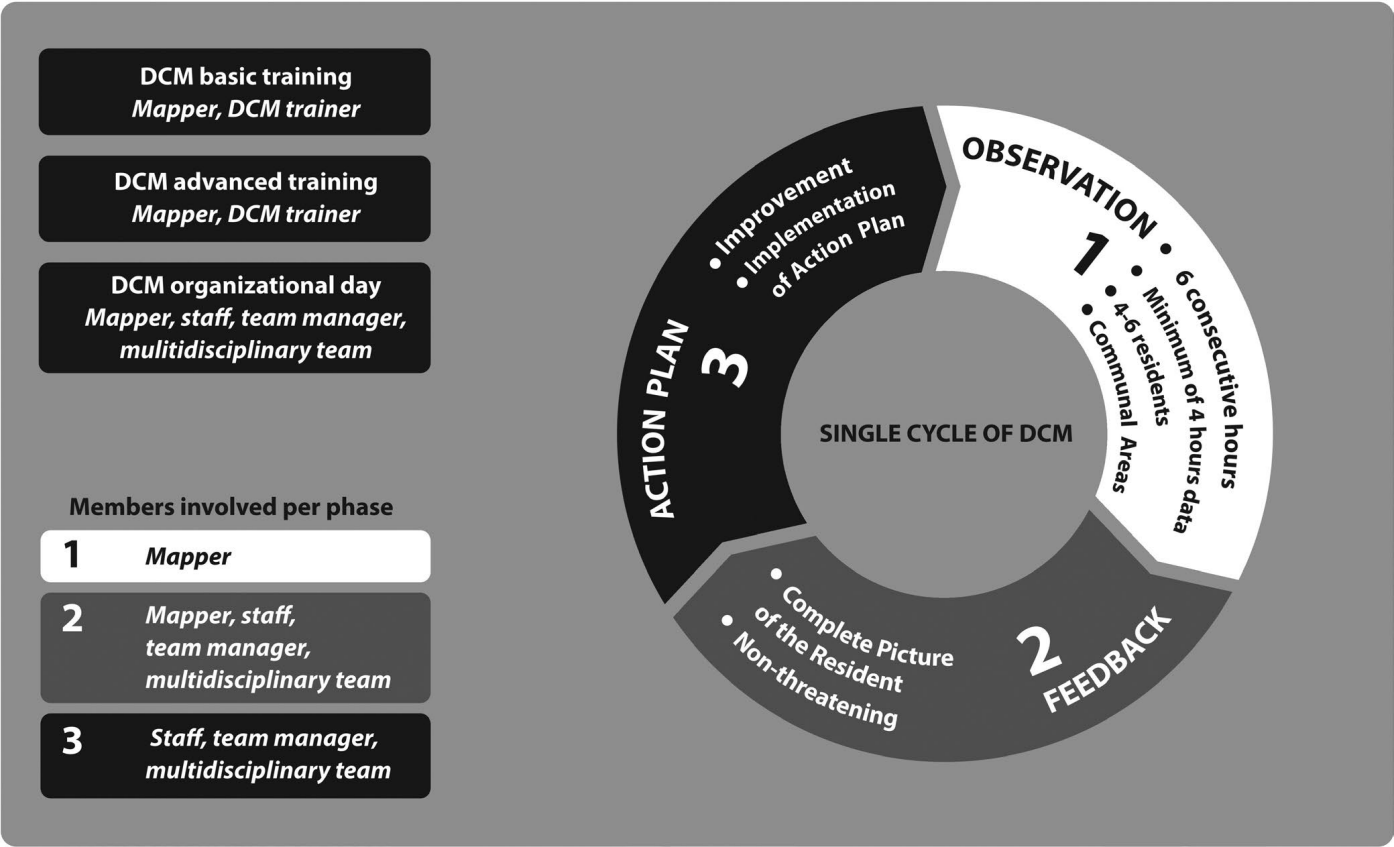
Dementia Care Mapping intervention components and cycle (based on: Van de Ven [2014])

Second, the mapper presented the results to all available staff and the manager of the group home in a report and in a feedback session focused on dementia and person‐centred care. Third, as part of the feedback, staff wrote up an action plan to improve support of their clients in daily practice. The action plans were explicitly discussed by the mapper in the feedback session after the next cycle. This provided opportunity for staff to reflect on their planned action in routine daily care (see Box 1 and Figure 1).

### 2.3 Procedure and sample

First, we approached all six major ID‐care organizations which had at least four group homes for older clients in the north of the Netherlands. Second, each organization provided four group homes for the study. A group home houses a small number (range 4–12) of older people with intellectual disabilities in need of care, support and supervision by care staff are living together. All participants were clients living in such small‐scaled group homes. The possibilities for using DCM determined our inclusion criteria for the group homes; we needed the possibility to observe four people simultaneously in a shared area (e.g., a living room) for at least two consecutive hours, the presence of at least three older people with (a strong suspicion of) dementia and a stable team not anticipating reorganization.

A complete cycle of DCM‐in‐ID was carried out twice in all group homes, with an interval of seven months, along with a quasi‐experimental study. To guarantee intervention adherence, the DCM trainers strictly monitored the intervention and supported the newly trained mappers in following the DCM‐in‐ID implementation protocol (Bradford Dementia Group, 2014). This protocol includes a description of all DCM‐preconditions and of every step needed to implement DCM in ID‐care (Bradford Dementia Group, 2014). This monitoring ensured that DCM was implemented and applied in all group homes in a similar way. We collected both quantitative and qualitative data among ID‐care staff from twelve group homes for older people. In each group home live a small number (range 6–12) of people with intellectual disabilities in need of care and support. Of these residents, 21% had a mild intellectual disability, 49% a moderate and 31% a severe or profound one. Regarding dementia, 46% had a diagnosis or a strong suspicion of dementia, and 18% showed some signs of dementia. The care and support deal with all aspects of everyday life, including activities of daily living (ADL) and day care activities.

The quantitative data regarded responses to questionnaires by care staff in each of the twelve group homes. From each group home, we included all staff with regular employment and excluded students doing an internship. Staff could fill in the questionnaire either on‐line or on paper. Data were anonymized by giving each staff member an identification number.

Qualitative data were obtained from two staff members per care facility (*N* = 24), as well as from all group home managers (*N* = 10), all behavioural specialists involved (*N* = 7) and all DCM‐ID mappers (*N* = 12). We conducted a total of eight focus‐group discussions, four after the first application of DCM and four after the second (Table 1). Staff members were invited at random for both time points. The discussions were categorized according to staff function: two regarded staff members from various group homes, one regarded managers and behavioural specialists, and one regarded all DCM mappers. The focus‐group discussions and individual interviews were semi‐structured, led by a discussion leader (FDS, GJD or EJF) accompanied by an observer (FDS, GJD and ASF), and by an interviewer (FDS), respectively. Individual interviews were held with participants who could not attend a focus‐group, four after the first cycle and two after the second cycle. The interviews and focus‐groups lasted approximately 1.5 hr, and were audio recorded and then transcribed in full.

**Table 1:** Background characteristics of the work setting

Staff	
*N*	136
Mean age in years (*SD*; range)	45 (12.4; 20–65)
Female (%)	90
Position	
Daily care professional (%)	63
Senior‐/coordinating care professional/personal coach (%)	32
Permanent employment (%)	90
Working hours/week (mean)	23
Education	
Elementary/secondary education (%)	9
Secondary vocational education (%)	80
Higher professional education (%)	11
Experience	
>11 years in ID‐care (%)	69
>11 years in current group home (%)	32
Training in person‐centred method(s) (%)^a^	84
Education in care for older people with intellectual disabilities (%)^b^	76

Work setting of staff in group homes	
Average number clients per group home (range)	9 (6–12)
Mean age of clients in years (*SD*; range)	67 (11.3; 34–92)
Clients with disability, by degree	
Mild (%)	21
Moderate (%)	49
Severe/Profound (%)	31
Clients with dementia	
Diagnosed (%)	35
Suspicion/Signs of (%)	29
Autism (%)	28
Other psychiatric diagnosis (%)	22
Challenging behaviour (%)	31
Severe behavioural problems (%)	5
Mobility/motor problems (%)	53
Communication problems (incl. sight and hearing) (%)	66

^a^ Method Urlings (Urlings, 2014), Validation (Bakken, Sageng, Hellerud, Kildahl, & Kristiansen, 2017), Reminiscence therapy (Van Puyenbroeck & Maes, 2008, 2009), Emotion‐oriented care (Finnema, Dröes, Ribbe, & Van Tilburg, 2000; Schrijnemaekers et al., 2002) and/or Gentle Care (Buijssen, 1991).

^b^ Training in methods named above or a self‐developed training by the organization.

### 2.4 Topics and measures

The quantitative data were derived from self‐developed open and closed questions for evaluating the use of DCM in daily ID‐care. We asked whether and to what extent DCM was a usable and practical addition to care. These questions were incorporated in follow‐up questionnaires in a quasi‐experimental trial. The qualitative data were gathered using the empathy map, derived from the methodology of the design‐thinking theory (Curedale, 2013). The empathy map facilitated tracing of the “pains and gains” of the participants, allowing them to discuss what they “think and feel,” “say and do,” “hear” and “see” about the first use of DCM in ID‐care. This method was developed to provide in‐depth information regarding opinions and experiences of participants.

### 2.5 Data analysis and reporting

We analysed and reported the data in three steps. We first described the background of the sample. We then reported on experiences and opinions of staff regarding the use of DCM in ID‐care after each DCM‐cycle. We finally reported on which factors influenced the evaluation of this method by staff, group home managers and DCM‐in‐ID mappers. We performed separate analyses for quantitative and qualitative data.

We analysed the questionnaire data on experiences and opinions of staff in response to the closed questions using descriptive statistics, with IBM SPSS statistics (version 25). We tested the differences between both measurements using Pearson's chi‐square tests. We analysed the qualitative data and open questions following the principles of conventional content analysis (Hsieh & Shannon, 2005) and thematic analysis (Braun & Clarke, 2006; Guest, MacQueen, & Namey, 2011), respectively, both with use of Atlas.ti computer software (version 7.5; Atlas.ti Scientific Software Development GmbH, Germany). After verbatim transcription of the contents of the focus‐group discussions and interviews, the first author (FDS) read and re‐read all transcriptions, set up a concept code book with initial codes and then discussed it with the second author (GJD). Next, both authors (FDS and GJD) coded and compared transcripts. Based on this comparison, we refined, relabelled and regrouped the codes until reaching consensus. We reported these results according to the themes derived from the data: understanding of the clients, professional reflection, knowledge and skills, organization of care and use in daily care. These themes provided the basis for analysing the open questions in the questionnaire. After coding all open responses, we divided the codes into the four themes, according to which we then reported the results.

## 3 RESULTS

### 3.1 Background characteristics

Of the 167 approached staff members, 136 (81%) have returned a questionnaire at both time points. Table 1 provides information on the background characteristics of the participating staff and on the group homes involved in the study. The participants were mostly (senior) daily care professionals (95%) with secondary vocational training (80%). All were relatively experienced staff (69% had over 11 years of experience) and most (84%) reported they received training in person‐centred care and in caring for older people with intellectual disabilities, respectively, 84% and 76%.

### 3.2 Opinions and experiences regarding DCM in ID‐care

The quantitative data indicated that the majority of all participants in the group homes (61% to 84%) agreed in both measurements with the statement that DCM is a good way to map clients’ behaviour, and provides new cues and insights for giving support to their clients (Table 2). About half of the participants (40% to 51%) agreed with the statements that DCM provided new insights into their work, that it helped to understand clients’ behaviour and that taking the perspective of the client was eye‐opening. This made them more conscious of their own behaviour towards their clients and helped them to provide better care. Furthermore, whereas after the first cycle of DCM 16% of the staff reported that DCM gave them more confidence in providing care for their clients, after the second cycle this was 31%, a significant increase. Moreover, after the second cycle of DCM the staff were even more positive about the benefits of DCM. However, staff stated that DCM did not influence their job satisfaction. Of the staff, 35% to 64% agreed with the statements that they worked in a more person‐centred way after DCM, and had gained more knowledge of dementia as well as more knowledge and skills for providing “good care.” Overall, at both time points 62% to 80% of all staff in the group homes found DCM a very usable and valuable addition in daily care. However, although the action plans were perceived as useful (68% to 76%), to maintain them in daily practice turned out to be difficult. Between 75% and 79% found DCM useable also in care for older residents without dementia.

**Table 2:** Opinions and experiences regarding DCM in ID‐care, in % (*N* = 136)^a^

	Measurement	Totally agree (%)	Agree (%)	Neutral (%)	Disagree (%)	Totally disagree (%)
Information about clients						
DCM is a good approach to map general behaviour of clients	1	9	72	19	1	0
	2	12	72	12	3	1
DCM is a good approach to map challenging behaviour of clients	1	9	63	25	4	0
	2	10	69	19	1	1
DCM is a good approach to map our way of providing care	1	4	64	29	2	1
	2	8	54	35	2	2
DCM provided me new insight in working with older people with intellectual disabilities	1	4	36	52	7	2
	2	6	45	35	13	2
Because of DCM I can better interpret residents’ behaviour	1	3	40	46	9	3
	2	6	35	47	11	1
Professional reflection						
Looking at care from the clients’ perspective was eye‐opening for me	1	3	36	33	19	10
	2	5	36	42	14	4
DCM made me more conscious of my interactions with the residents	1	5	50	34	9	2
	2	10	47	33	9	1
DCM helped me provide better care to the residents	1	6	39	47	8	1
	2	8	40	41	12	1
DCM made me feel more confident in providing care to my clients*	1	3	13	65	15	5
	2	4	27	52	16	1
DCM gave me more job satisfaction	1	1	7	66	20	6
	2	2	9	66	19	5
Knowledge and skills						
DCM made me work in a more person‐centred way	1	4	31	50	12	2
	2	8	35	43	13	1
DCM gave me more knowledge of dementia	1	2	35	42	21	1
	2	1	44	39	13	2
DCM provided tools for providing “good care”	1	5	50	40	6	0
	2	7	58	29	6	1
Organization of daily care						
DCM is a valuable addition to the methods we are currently using	1	6	56	30	8	0
	2	10	56	29	5	0
Discussing clients with the whole team had added value for me	1	15	65	20	0	0
	2	17	61	20	1	1
Discussion with the whole team provided new insights	1	7	64	27	3	0
	2	13	62	19	5	1
I find DCM‐action plans supportive in daily care	1	8	60	27	4	0
	2	9	63	26	2	1
Use in daily care						
I find DCM useable in daily care	1	5	59	33	3	0
	2	9	61	26	3	1
DCM is also useable in care for residents without dementia	1	4	68	25	0	0
	2	14	65	19	1	1
The DCM report has little added value for me	1	1	10	39	42	10
	2	0	12	43	33	12
Maintaining the action plans was not practicable	1	3	19	58	18	4
	2	1	20	48	27	4

^a^ Due to rounding the results could add up from 99 to 101.

* Significant difference between measurements 1 and 2 (*p* = .048).

### 3.3 Underlying factors

The factors underlying staff experiences and opinions were derived from the qualitative data and the open questions in the questionnaire. Table 3 describes participants in the focus‐group discussions and personal interviews. The results of staff experiences in the use of DCM in ID‐care from a professional perspective were reported per theme as derived from the qualitative data: information about clients, professional reflection, knowledge and skills, organization of care and use in daily care. The following paragraph will elaborate on these topics.

**Table 3:** Participants in focus‐group discussions (FGD) and individual interviews (IV)

	First measurement		Second measurement	
	FGD	IV	FGD	IV
Nr. of FGD/IV	4	4	4	3
Nr. of participants				
Mappers^*^, ^a^	12		9	3
Staff	14	2	13	3
Group home managers^*^, ^a^	5	5	7	
Behavioural specialists^*^, ^a^	2		5	

^a^ Participants took part in both measurements, whether in a focus‐group discussion or an interview.

### 3.4 Information about clients

We found the most dominant underlying factor for the experiences with and opinions on DCM to be the degree of insight which DCM provided regarding the causes of specific client behaviours. Examples were behaviour caused by over‐ or under‐responsiveness, physical discomfort (cold, inappropriate furniture and aids) and (behavioural) consequences of dementia. Especially, the under‐responsiveness of and the lesser attention to quiet and non‐demanding clients were striking to some staff. Staff highly appreciated DCM’s accurate and detailed mapping of the behaviour, which gave them confidence in the method. Staff reported that the feedback of the mapping made them more alert to clients’ behaviour and needs. Furthermore, the mapping and feedback helped staff to connect current challenging behaviour to clients’ histories. Staff also gained insight into the influences of external and environmental factors on the behaviour of clients (opening and closing doors, client sitting alone, client getting no attention and many staff passing by), and on interaction with other clients. Moreover, staff reported that DCM changed their perspective on optimal care and led to a different way of working, which, in turn, affected the behaviour of the clients. They mentioned that clients were more at ease and that the groups had a calmer pace during the day. Nevertheless, staff and managers mentioned that two cycles of DCM were too short to confirm a decisive difference in behaviour of clients. They did, however, expect to see an effect after a longer, more routine application of DCM.

I thought, through my experience and all other courses, that it was important to divide my time and attention over everyone, and not just to the demanding residents or residents with challenging behaviour. After being observed with DCM I have noticed that I do not do that right. Whereas I thought I was doing it very purposely. (Staff 6.2)

You look at it very differently. You are more critical. When I look at client J., I often let him colour. I thought he liked it, but DCM showed that J. likes colouring at first, and then he just goes on because he has a pencil, but he does not like it anymore. DCM provides a lot of awareness, so that you also offer something different. You change activities, think about it consciously. (Staff 1.2)

I noticed that we talk a lot in the team meetings about the residents with problems. Through DCM we noticed that by observing those without problems also a lot of profit is to be gained. Someone just sits in the room and does nothing. We became aware of this through DCM. We can then offer something. And indeed, when you offer something, you also have to enable the person to stop again, because we forgot that. (…). There is more to do than focus on those who cause problems. (Staff 3.5)

The biggest thing we noticed is the great influence of staff on the mood of the clients. From our observation it became very clear that when staff leave the group the dynamics change completely. And you notice the frustrations of employees who say: if we could only be more present, then that client would be more at ease. Would have fewer negative interactions with his neighbour client. We knew that, but it was confirmed again. (Manager 5.1)

### 3.5 Professional reflection

Staff, managers and mappers reported that DCM improved their professional reflection, thereby providing a basis for a different care approach. The mapping and feedback sessions made them increasingly aware of their own professional behaviour and the effect of their interactions with clients. The open questions and the qualitative data indicated agreement among staff that DCM creates awareness by mirroring their professional behaviour, thereby providing a base for change in their behaviour. It made them aware of their own blind spots in their care and interactions, and in dealing with the behaviour of their clients. This led to more consciousness and alertness. Furthermore, they became more aware of the conscious and unconscious influence of staff members on both clients and group processes, and the resulting staff‐client behaviour and interactions.

Yes, more consciously. That you focus on one client and take the time for it. Do not fly past, things like that… just walk, don't fly by, but adjust your pace. (Staff 3.2).

What they told me in the team was that it was so helpful that an external person came to observe, who could also comment on the blind spots of staff. This became very clear in the feedback. Observation is, in my opinion, one of the most powerful things we have. Just watch: what is happening to him? And then get out of your ordinary habits. Because the employees are all doers. And they are all used to certain behaviour of their client, but they do not know what is behind it. I liked it so much: one of our clients was always fiddling with her hands. Employees know that this is happening, but not under what circumstances and why so often and how it affects the client, and why she does it. That emerged because of DCM, and we had a discussion about it, and then we could do something with it. (Manager 3.1)

A number of outcomes from the observations were very practical, things like: 'client cannot reach the floor with her feet’, for example. There is a lot of waffling after that observation, like: ‘What is wise? Should we do something or not do something? Why is that client sitting there? And if she has an adjusted chair there, then…’ So it caused a lot of discussion in the good sense of the word. And not everything can be solved right away, but at least the consciousness of 'gee, we didn't even notice that she cannot reach the floor with her feet'. Just as you said, that fiddling. (Manager 5.2)

### 3.6 Knowledge and skills

In general, DCM provided more knowledge and skills on ageing and dementia, and on person‐centred care. Staff indicated that they knew better what they were doing in their work, and why. According to staff, managers and mappers, this resulted in more person‐centred, deliberate and in‐depth care, which was highly appreciated. DCM gave them more ground in providing care; they were able to relate care to the needs of the client, based on the theory of person‐centred care, as well as to the consequences of ageing and dementia. Staff reported that by making the needs of the clients visible using Kitwood's five dimensions of personhood (Kitwood, 1992) improved the interpretation and explanation of clients’ behaviour. This was reflected in the action plans; the proposed actions had a theoretical basis in person‐centred care and knowledge of dementia. However, staff experienced a conflict between person‐centred working and the task‐oriented organization of care and the registration systems.

Staff who indicated not having gained more knowledge of dementia did confirm that DCM helped them to put into practice the knowledge they had gathered from prior courses. Staff and managers stated that where (person‐centred/dementia care) knowledge was often latently present, DCM provided the tools to put this knowledge into practice.

DCM makes you think about: what are you doing, why do you do this and what needs of the client does this meet? (Staff 6.8)

DCM provides a practical dimension [to prior knowledge ‐ FS] (…). Employees said: 'yes, we work in a person‐centred way’, but what does that mean in daily practice? They might do it in their heads, I guess. And I also know that staff are convinced that they do it. But DCM shows how it is on all levels of care, and whether it is true. (…) But it also helps in discussing how we are working together. But then, it does not last. And staff say: ‘but we had a course'. Then I think: 'What of it has remained in practice after a year?' DCM helps to put it into practice so that it lasts. (Manager 1.1)

I think a nice thing about this method is that you really start at baseline. You identify certain things, and then you can work on expertise. We are used to throwing in a method or a training, and then getting started. And now [with DCM ‐ FS] you start with: how do we do things? And because of actively involving staff, a solid ground is created to continue working with existing methods. That all still needs to be done, but I think this a very good start. (Manager 5.1)

### 3.7 Organization of daily care

According to most staff and managers, DCM helped to improve the organization of daily care. In the qualitative data and open questions of the questionnaire, they reported that the method created more mutual exchange within the teams, more coordination and conformity of care and a greater feeling of being a team. Staff were not aware that they used different approaches which may have caused confusion in clients. Staff mentioned that they tend to work in a habitual, task‐driven way. The exchange within the teams stimulated staff to come up with a mutual action plan for both individual clients and the group, in consultation with the behavioural scientist and the manager but not being guided only by the ideas of the behavioural scientists. Staff and managers reported that this brought more responsibility, greater depth and more deliberate actions of staff into daily care. This was appreciated by most staff, although some found it difficult to deal with the responsibility as well as the reflection on their own actions in daily care. Most greatly appreciated the systematic, methodical and repetitive cycle of DCM, because this had been insufficiently present in routine daily care.

The actions are very practical, because you learn to take the perspective of the client. What that person needs. That is what we discuss, and then we try to do the same. (Staff 2.3)

She [the mapper – FS] really knew how to get the conversation going, to ask critical questions. That was a major profit for the team, to get in touch with each other in that way. And they feel a need to continue together like that. That could indicate how the team normally works, but yes, this certainly added something for them. What they also found out within the team is that they now do the morning rituals very differently, they coordinate a lot more. Like: ‘Gosh, how do you do that in the morning with that client? How do you get him out of bed?’ That is now being discussed much more clearly. And also adapted to each other, and agreed that we are going to do it like that now. (Manager 2.1)

DCM is a lot more methodical, with clear methodical steps. Other courses and approaches don't have that; these are often more general visions. This is not contradictory; DCM maps what a situation looks like and goes into ‘what to do then’? And then you go into what you don't know, which is more diagnostic. (Manager. 3.2)

I found it [DCM ‐ FS] very different from what we normally did automatically, yes, certainly complementary. What we normally did when we saw challenging behaviour was: look at the methods, at vision or at courses or training, and then we would look at what we do in. With DCM we start with what happens in daily practice, and then apply what we have learned. It is just very practical. (…) For the team it was really a very pleasant way of working. Talking in a very different way. (…) It immediately gave direction and clear advice. That was really nice. (Manager 3.1)

### 3.8 Use of DCM in daily care

Staff, managers and mappers reported that they found DCM very practical and applicable. In the qualitative data and open questions, they almost unanimously agreed that they would like to continue DCM in daily care and recommend it to other, comparable, organizations. However, for maintenance and implementation, participants suggested some improvements. First, the action plans should be incorporated in the personal plans of the clients and in the registration systems. However, these are not matched in the organization of care, according to staff and managers. Second, the action plans should become a standard part of the team meetings. Third, the managers and the behavioural specialists should provide support in setting up attainable action plans, because some staff found it difficult to set up such plans themselves. Finally, staff wanted to use DCM more periodically, for example each half year and in cases of new clients in the group home, and if possible, also for individual observations, so as to focus more on problems in private areas, like assisting individual residents in activities of daily life (ADL).

The conversation becomes easier. Especially in case of difficult decisions or differing opinions. With DCM the focus is no longer on the diverse opinions and perspectives of everyone around the client, but the needs of the person in question are the starting point. (DCM‐in‐ID‐mapper 4.1)

For us it works well: the staff member reports the things indicated by DCM. That may be that someone is always very lonely, or someone who seems very easy gets a bit neglected. A goal‐oriented report was made by the personal coach; at least three times a day an attention moment was given, besides the regular care moments. And then you could score whether you had done that… If you see that your colleague has done a certain thing, then you think 'Oh, that is actually very nice', but you also notice yourself thinking, 'I also can't say I did nothing’. It encourages you to do something yourself. ( 4.2)

Yes, we have something to do with that. You have to keep it alive, at least I do. It is a method that is being used, certain things have pointed out and have to be implemented in practice. Staff have to get used to that. It is not all clear and easy and ready to be immediately put into practice, it does not work like that. It's a translation, of course. It is an attempt. (…) But the question is how to keep that alive? The action is clear and must be translated into practice. (manager 2.2)

It strikes me that at our location, DCM really has been an eye‐opener; we have to do much more with policies regarding care for older people; it has to be much more structured in the organisation. And many more DCM‐mappings, many more things need to be developed in terms of the policy to provide good care for older people. DCM is a great part of that; that you can see. (Manager, 5.1)

### 3.9 Comparison of results of both measurements

Comparison of the data from both measurements showed that after the second cycle of DCM staff were even more positive about the benefits of DCM (Table 2). We found both in the quantitative and qualitative data that the answers of staff given after the first cycle were aimed mostly at the clients and their behaviour. After the second DCM‐cycle, staff spoke more about their own professional reflection. This was also the case regarding the provision of person‐centred care. After the first cycle, staff were quite convinced that they worked in a person‐centred way. After the second cycle, staff agreed even more that this could be improved. They reported having become more aware that DCM is not an instant solution, but that they had to contribute themselves. They remarked that provision of care became more in line with the well‐being and needs of the clients, rather than task‐driven or habitual. However, this is not yet compliant with the organization of daily care, according to staff. Next, the multi‐organizational focus‐groups we observed that care staff found it inspiring and helpful to hear about and learn from each other's experiences. Furthermore, we found that in group homes with staff experienced in person‐centred care (f.i. using method Urlings), DCM was more successful. Finally, we found that staff belonging to one group home did not find DCM to have added value next to existing methods because of the mapper's way of providing feedback.

## 4 DISCUSSION

With this mixed‐method study, we examined the experiences and opinions of staff and group home managers on the use of DCM in ID‐care. In general, we found that professionals valued DCM positively in the care for older people with intellectual disabilities, with or without dementia. The method provided insights into the behaviour of clients, enabled professional reflection, provided new knowledge and skills regarding dementia and person‐centred care, and helped to apply this theoretical knowledge in practice. However, we found that not all group homes completely fulfilled the DCM‐preconditions which had previously been found to be successful (Schaap, Dijkstra, et al., 2018). Finally, we found that staff appreciated DCM even more after the second cycle than after the first.

The quantitative data indicated that the majority of participants found DCM a usable and valuable addition to daily care. It provided new insights into clients’ behaviour and into their own professional behaviour, and gave new cues for organization of care. Furthermore, almost the half of the staff reported having gained more knowledge and skills for dementia‐ and person‐centred care which had slightly increased after the second DCM‐cycle. This indicates that staff, who in majority stated to have had previous training in dementia and in person‐centred methods, were enabled by DCM to apply this knowledge better. Moreover, DCM was applied for a relatively short time, a longer follow‐up period may be useful, as a transition to more person‐centred care may require more time than provided by the follow‐up of our study (see also Schaap et al., 2019b). However, staff stated that DCM did not influence their job satisfaction, a result also found in previous research: feedback as provided showed DCM to be helpful and possibly leading to enhanced job performance (Visscher, Peters, & Staman, 2010), but showed job performance hardly to have affected job satisfaction (Bartlett, 2001; Squires et al., 2015).

We found, first of all, that the most frequently mentioned underlying factors for positive experiences with DCM in ID‐care were that DCM increased insights both into the behaviour of both clients and professionals. The insight into clients, related to new knowledge regarding dementia and person‐centred care, led to more understanding of the causes of their behaviour and ideas for more tailored care. Furthermore, DCM improved professional behaviour; the method enabled professional reflection and provided guidance in ID‐care, which we found had been uncommon for daily ID‐care staff. These factors may contribute to coping with challenges in long‐term care. Our study and previous ones indicated that long‐term care relationships are important for understanding the behaviour of clients, but can also cause blind spots and impede a critical look at one's own professional behaviour (Bekkema, de Veer, Hertogh, & Francke, 2015; Donaldson & Grant‐Vallone, 2002; Finkelstein, Bachner, Greenberger, Brooks, & Tenenbaum, 2018; Iacono et al., 2014; Janssen & Van der Vegt, 2011; Murray, 2005). Previous research showed that strong bonds with clients and high engagement with work may lead to ID‐care staff taking on overly demanding responsibilities and refusing to admit mistakes in daily work (Donaldson & Grant‐Vallone, 2002; Janssen & Van der Vegt, 2011; Murray, 2005). Moreover, previous studies found that professional reflection and understanding are important to overcome this habitual professional behaviour and these blind spots; such reflection could lead to improved job performance (Eccles et al., 2012; Potthoff et al., 2018; Presseau et al., 2014; Visscher et al., 2010). We found that DCM helped to achieve this because it provides recurring feedback and reflection on job performance, in combination with greater knowledge regarding dementia and person‐centred care: factors not yet common in ID‐care (Fokkens et al., 2016).

A second value of DCM seems to be its provision of new knowledge and skills regarding dementia like person‐centred care, cues to coordinate care and a methodical tool to apply knowledge in practice; these have not been reported for any other method in ID‐care. This report by professionals contradicts the large number of existing approaches for providing care and support to people with intellectual disabilities (Singh, 2016a, 2016b; Twint & Bruijn, 2014), which often lack either a theoretical, scientific or methodical base (Fokkens et al., 2016; Maaskant, Balsters, & Kersten, 2017). We found that DCM provided an underlying, person‐centred, theory for staff in daily care provision by relating the needs of clients to Kitwood's five dimensions of personhood. This led to more deliberation of individual staff in daily care, which are factors associated with improved job performance (Chiniara & Bentein, 2016; Maurits, de Veer, Groenewegen, & Francke, 2017; Squires et al., 2015). Staff reported that the methodical cycle helped them to sustain the application of theoretical knowledge on person‐centred care, ageing and dementia in practice, and to bridge the gap between knowing and doing, as also shown in previous research (Grimshaw, Eccles, Lavis, Hill, & Squires, 2012; Slaughter et al., 2018). Furthermore, the improved coordination and conformity of care provided by DCM created in staff a feeling of being a team, which previous research has also shown to be an enabling factor for providing good care (Fyffe, McCubbery, & Reid, 2008; Kersten, Taminiau, Schuurman, Weggeman, & Embregts, 2018). DCM thus provides an applicable theory for the provision of daily care.

Third, staff reported that DCM helped to apply a more person‐centred approach, which was perceived as helpful to fulfil the individual clients’ needs. However, staff experienced a conflict between person‐centred working and the task‐oriented organization of care and registration system. This conflict was also noted in previous studies on use of DCM in nursing homes and was identified as an important barrier for its implementation (Kadri et al., 2018; Griffiths et al., 2019; Van de Ven et al., 2014). Several studies have shown that a person‐centred point of view should be the guiding principle in providing high‐quality care for older people with intellectual disabilities, but most ID‐care organizations have a task‐oriented organization of care (Cleary & Doody, 2017a, 2017b; Holst et al., 2018; Ouellette‐Kuntz et al., 2019).

To be successful, DCM requires fulfilment of preconditions, which we found were not always present, such as a strong base of person‐centred care throughout the organization. Previous research on DCM indicated that to reach optimal results the fulfilling of these preconditions is of major importance (Quasdorf et al., 2017; Schaap, Fokkens, et al., 2018; Schaap, Dijkstra, et al., 2018; Van de Ven et al., 2014). For future implementation of DCM, adequate compliance to the preconditions should be maximized.

Finally, we found that staff were more positive about DCM after the second cycle. This has also been shown in previous research regarding its use in dementia care (Quasdorf & Bartholomeyczik, 2017; Van de Ven et al., 2014). This could be for several reasons: first, the DCM‐in‐intellectual disabilities mappers had become more experienced in carrying out DCM and were therefore able to provide better feedback. Second, staff had become more aware of what DCM entails: not instant solutions, but reflection on professional behaviour and finding solutions themselves. Previous research showed that this mechanism is common after implementing a new intervention; participants have to become used to working with it (Grilo, Santos, Rita, & Gomes, 2014; Kersten et al., 2018; Slaughter et al., 2018; Wood et al., 2014). Related to this is that the answers of staff after the first DCM‐cycle were aimed mostly at (the behaviour of) clients, but after the second cycle they were aimed at reflection on their own professional behaviour. Previous research showed that when not in control, staff are inclined to attribute problems in daily care to the clients’ behaviour (Farrell, Shafiei, & Salmon, 2010; Squires et al., 2015). This suggests that after the second DCM‐cycle, staff are used to doing more professional reflection and are more in control of their daily work, which may lead to improved job performance.

### 4.1 Strengths and limitations

A key strength of this study was our use of a multi‐informant and multi/mixed‐method design to examine the opinions of care staff and managers on DCM in ID‐care settings. Moreover, we examined the use of DCM in practice in 12 group homes from six organizations for people with intellectual disabilities, each home having its own vision, culture, team characteristics and habits in care; this enhances the validity of our results for routine ID‐care practice.

A first limitation of this study is that we rely fully on reports by professionals. These reports may be biased due to social desirability and a Hawthorne effect, related to the additional attention to professionals as part of the study. However, this is not very likely because the methods used in the interviews and focus‐groups enabled staff to perform critical reflection and take part in in‐depth discussion. This makes a Hawthorne effect not very plausible, although it cannot be ruled out. Second, we used a self‐developed questionnaire which had not been validated. Third, another limitation might be that the observation, report and feedback were carried out by a DCM‐in‐ID mapper from an external organization. However, a feasibility study of DCM in ID‐care and this study showed that the fresh look of an external person improved independence and led to avoidance of interpretation bias due to familiarity with habits, clients and colleagues (Schaap, Fokkens, et al., 2018). Finally, regarding performance quality, that is the mappers’ skills: despite finishing the basic and advanced mappers’ trainings, the newly trained mappers were not always fully experienced in providing feedback. This may have caused a lower quality of the feedback and the further results.

### 4.2 Implications

Our finding that DCM provides new knowledge and skills for staff caring for older people with intellectual disabilities could bridge the gap in the changing approach to care for this group (Chapman, Lacey, & Jervis, 2018). The method was perceived as useful for applying theoretical knowledge in practice, also knowledge gained from previous training and courses. However, for a routine application of DCM a broader (theoretical) knowledge on the part of staff in person‐centred care should be considered. Moreover, to enable staff to provide more person‐centred care, a shift would be advisable towards person‐centred care throughout the whole organization, a shift from a task‐oriented way of working to a person‐centred compliant vision.

Although we found that staff valued DCM in daily care practices and that indications that it might improve job performance and quality of (person‐centred) care for older people with intellectual disabilities, previous research has found no evidence on the quality of life of people with intellectual disabilities nor on job satisfaction of care staff (Schaap et al., 2019a, 2019b). The effectiveness of DCM should thus be assessed in a study aimed at outcomes in the direct care process, such as job performance, quality of (person‐centred) care and quality of staff–client interactions.

## 5 CONCLUSION

Staff considered the use of DCM in ID‐care to be a valuable additional method to support them in their work with ageing clients. We found that DCM gave insights and consciousness, new knowledge and skills, a (person‐centred) theoretical base and a methodical cycle to sustain knowledge in practice. This indicated that DCM could improve the quality of care and job performance of staff. However, the implementation and maintenance of DCM need further attention, as does compliance to the action plans. Future research should follow‐up on the effects of DCM in ID‐care on quality of care, quality of staff‐client interactions and job performance.

## CONFLICT OF INTEREST

The authors have no conflict of interest to declare.
